# Value-Added Compounds with Antimicrobial, Antioxidant, and Enzyme-Inhibitory Effects from Post-Distillation and Post-Supercritical CO_2_ Extraction By-Products of Rosemary

**DOI:** 10.3390/antiox12020244

**Published:** 2023-01-21

**Authors:** Simon Vlad Luca, Gokhan Zengin, Kouadio Ibrahime Sinan, Izabela Korona-Glowniak, Mirjana Minceva, Krystyna Skalicka-Woźniak, Adriana Trifan

**Affiliations:** 1 Biothermodynamics, TUM School of Life Sciences, Technical University of Munich, 85354 Freising, Germany; 2 Physiology and Biochemistry Research Laboratory, Department of Biology, Science Faculty, Selcuk University, 42130 Konya, Turkey; 3 Department of Pharmaceutical Microbiology, Faculty of Pharmacy, Medical University of Lublin, 20-093 Lublin, Poland; 4 Department of Natural Products Chemistry, Medical University of Lublin, 20-093 Lublin, Poland; 5 Department of Pharmacognosy and Phytotherapy, Faculty of Pharmacy, “Grigore T. Popa” University of Medicine and Pharmacy Iasi, 700115 Iasi, Romania

**Keywords:** *Rosmarinus officinalis*, *Salvia rosmarinus*, aromatic herbs, wastes, residues, LC-HRMS/MS, essential oils

## Abstract

Hydrodistillation is the main technique to obtain essential oils from rosemary for the aroma industry. However, this technique is wasteful, producing numerous by-products (residual water, spent materials) that are usually discarded in the environment. Supercritical CO_2_ (SC-CO_2_) extraction is considered an alternative greener technology for producing aroma compounds. However, there have been no discussions about the spent plant material leftover. Therefore, this work investigated the chemical profile (GC-MS, LC-HRMS/MS) and multi-biological activity (antimicrobial, antioxidant, enzyme inhibitory) of several raw rosemary materials (essential oil, SC-CO_2_ extracts, solvent extracts) and by-products/waste materials (post-distillation residual water, spent plant material extracts, and post-supercritical CO_2_ spent plant material extracts). More than 55 volatile organic compounds (e.g., pinene, eucalyptol, borneol, camphor, caryophyllene, etc.) were identified in the rosemary essential oil and SC-CO_2_ extracts. The LC-HRMS/MS profiling of the solvent extracts revealed around 25 specialized metabolites (e.g., caffeic acid, rosmarinic acid, salvianolic acids, luteolin derivatives, rosmanol derivatives, carnosol derivatives, etc.). Minimum inhibitory concentrations of 15.6–62.5 mg/L were obtained for some rosemary extracts against *Micrococcus luteus*, *Bacilus cereus*, or *Staphylococcus aureus* MRSA. Evaluated in six different in vitro tests, the antioxidant potential revealed strong activity for the polyphenol-containing extracts. In contrast, the terpene-rich extracts were more potent in inhibiting various key enzymes (e.g., acetylcholinesterase, butyrylcholinesterase, tyrosinase, amylase, and glucosidase). The current work brings new insightful contributions to the continuously developing body of knowledge about the valorization of rosemary by-products as a low-cost source of high-added-value constituents in the food, pharmaceutical, and cosmeceutical industries.

## 1. Introduction

Aromatic herbs/plants are important constituents of human nutrition, valued for their aroma, flavor, and color, as well as their preservative and nutraceutical properties. Moreover, these plants are acknowledged to contain a wide range of bioactive metabolites that make them promising drug lead candidates to treat numerous human ailments [1]. Among aromatic herbs, rosemary (*Salvia rosmarinus* Schleid., formerly *Rosmarinus officinalis* L.) has attracted particular attention over the years due to its culinary, cosmeceutical, and pharmaceutical uses [2]. Rosemary leaves have been used in traditional folk medicine to treat dysmenorrhea, muscle spasms, and renal colic [3]. Phytochemical studies have evidenced the abundance of volatile terpenes usually isolated as essential oils (EOs), such as *α*-pinene, eucalyptol, camphor, bornyl acetate, and eugenol. Further data have also indicated the presence of flavonoids (e.g., luteolin, hesperidin, diosmin, genkwanin), phenolic acids (e.g., rosmarinic acid, chlorogenic acid), diterpenes (e.g., carnosol, carnosic acid, rosmanol, rosmadial, rosmaquinones), and triterpenes (e.g., oleanolic acid, ursolic acid) in solvent extracts [4,5]. These constituents are assumed to be responsible for a plethora of bioactivities, such as antioxidant, anti-inflammatory, anti-ulcer, cardiovasculo-protective, neuroprotective, hepatoprotective, and anticancer effects [4,5,6]. Moreover, rosemary is also used in cosmetic formulations to treat ultraviolet oxidative damage, cellulite, alopecia, and aging [3].

The extraction of EOs represents one of the main reasons for the large-scale cultivation of rosemary. Steam and hydrodistillation remain the major technologies that satisfy industrial needs for rosemary EO due to their low cost, simplicity, and generation of high-quality oils [7]. However, various post-distillation by-products, including spent plant materials (solid residues), aqueous condensates (hydrolates, hydrosols), and residual waters (distillation waters or leachates), are generated in large amounts. Usually, these by-products are considered wastes and discarded in the environment without further processing [8]. Literature data on the recovery of post-distillation by-products from rosemary are scarce. For instance, Irakli et al. [2] developed a liquid chromatography method coupled with mass spectrometry to identify various phenolic compounds from a spent material extract of rosemary. Bouloumpasi et al. [9] explored the antioxidant and antibacterial properties of the material after steam distillation, whereas Yagoubi et al. [10] showed that rosemary distillation residues could reduce the lipid oxidation, increase the alpha-tocopherol content, and improve the fatty acid profile of lamb meat.

Supercritical CO_2_ extraction (SC-CO_2_) is an emerging technology alternative to steam or hydrodistillation that can provide several operational advantages. For instance, the preservation of thermosensitive terpenes is ensured as low temperatures and reduced extraction times are applied. SC-CO_2_ is widely accepted by the food, cosmetic, and pharmaceutical industries as a green solvent since its complete removal at the end of the process can be achieved without additional energy consumption [11,12]. SC-CO_2_ was briefly explored to extract volatile terpenes from rosemary [13,14,15]. Compared to steam and hydrodistillation, which generate multiple categories of by-products, SC-CO_2_ extraction produces only the spent plant material as the primary residue. The utilization of post-SC-CO_2_ by-products did not constitute the focus of previous works. Scrutiny of the literature retrieved one study that used the solid residues obtained after the SC-CO_2_ extraction of *Melissa officinalis* L. for the further extraction of phenolic compounds [16].

This study aimed to provide a comparative assessment of the phytochemical profile and biological potential of raw (EO, SC-CO_2_, and total extracts), post-distillation, and post-SC-CO_2_ extracts. Thus, the EO and SC-CO_2_ were profiled by gas chromatography coupled with mass spectrometry (GC-MS), whereas the remaining extracts were analyzed by liquid chromatography coupled with high-resolution tandem mass spectrometry (LC-HRMS/MS). A panel of Gram-positive and Gram-negative bacteria as well as yeast was used to evaluate the antimicrobial activity. The antioxidant activity was evaluated concerning the 1,1′-diphenyl-2-picrylhydrazyl (DPPH), 2,2′-azino-bis(3-ethylbenzothiazoline) 6-sulfonic acid (ABTS), cupric ion-reducing antioxidant capacity (CUPRAC), ferric ion-reducing antioxidant power (FRAP), metal chelating ability (MCA), and phosphomolybdenum (PBD), whereas the enzyme-inhibitory potential was assessed in acetylcholinesterase [17], butyrylcholinesterase (BChE), tyrosinase, amylase, and glucosidase tests.

## 2. Materials and Methods

### 2.1. Chemicals

Alkane standard solution (C8–C20, ~40 mg/L each, in hexane), 2′-azino-bis(3-ethylbenzothiazoline-6-sulphonic acid) (ABTS), 5,5-dithio-bis(2-nitrobenzoic) acid, acarbose, acetonitrile, ammonium acetate, ammonium molybdate, amylase (EC. 3.2.1.1, from porcine pancreas), *α*-bisabolol, butyrylthiocholine chloride, caffeic acid, *β*-caryophyllene, cupric chloride, 1,1-diphenyl-2-picrylhydrazyl (DPPH), eel acetylcholinesterase (AChE, type: VI-S, EC 3.1.1.7), ethanol, ferric chloride, ferrous sulfate hexahydrate, ferrozine, Folin–Ciocalteu reagent, formic acid, galantamine, acetylthiocholine iodide, gallic acid, glucose, glucosidase (EC. 3.2.1.20, from *Saccharomyces cerevisiae*), *α*-humulene, horse serum butyrylcholinesterase (BChE, EC 3.1.1.8), hydrochloric acid, hydroxybenzoic acid, kojic acid, limonene, *β*-myrcene, Mueller–Hinton (MH) broth, rosmarinic acid, rutin, sodium carbonate, sodium hydroxide, sodium molybdate, sodium nitrate, 2,4,6-tris(2-pyridyl)-s-triazine, Trolox, ethylenediaminetetraacetate (EDTA), and tyrosinase (EC1.14.18.1, mushroom) were from Merck KGaA (Darmstadt, Germany). *β*-Caryophyllene oxide was from Thermo Scientific (Olching, Germany), *α*-humulene was from Biomol (Hamburg, Germany), and liquid CO_2_ (≥99.7%) from Westfalen AG (Münster, Germany). Methanol was bought from VWR Chemicals (Ismaning, Germany). Acetonitrile and formic acid were from Avantor (Gliwice, Poland).

### 2.2. Plant Material

Dried leaves of rosemary were bought from a local market in Germany; the plant material was authenticated by one of the authors (A.T.). A voucher specimen (RO/220714) was deposited in Biothermodynamics, TUM School of Life Sciences, Technical University of Munich, Freising, Germany.

### 2.3. Extraction

#### 2.3.1. Preparation of Essential Oil

The powdered dried rosemary leaves (50 g) were placed in a Clevenger-type apparatus with 500 mL of deionized water and distilled for 4 h. The obtained rosemary essential oil (**REO**) fraction was collected and dried over anhydrous sodium sulfate. The hydrodistillation procedure was performed in duplicate.

#### 2.3.2. Preparation of SC-CO_2_ Extracts

The SC-CO_2_ extractions were performed on a Spe-ed SFE Zoran Extractor (Applied Separations, Allentown, PA, USA) which could operate at a maximum temperature and pressure of 180 °C and 690 bar, respectively. The extraction vessel was loaded with 40 g of powdered dried rosemary leaves, which were compressed to a bed length of 12.0 cm and a diameter of 3.0 cm. The vessel was sealed, placed in a thermostatic mantle, and connected to the extractor. The extraction was started after an equilibration static time of 10 min and performed for 30 min at a constant CO_2_ flow (7 standard liter min^−1^). All SC-CO_2_ experiments were conducted at a pressure of 100 bar, whereas the temperature conditions were 40 °C, 50 °C, and 60 °C, yielding **RC1**, **RC2**, and **RC3**, respectively. Each experimental setup was performed in duplicate.

#### 2.3.3. Preparation of Total, Spent, and Residual Water Extracts

At the end of the hydrodistillation process, the residual water was filtered and freeze-dried, affording the rosemary water extract (**RWE**). Totals of 10 g of the powdered dried rosemary leaves, spent plant material after hydrodistillation, and spent plant materials after the three SC-CO_2_ extractions were separately extracted at room temperature in a Bandelin Sonorex Digitec ultrasound bath from BANDELIN Electronic GmbH & Co. KG (Berlin, Germany) with 3 × 100 mL methanol/water 75/25 (*v*/*v*) in three repeated ultrasound cycles (35 Hz), each 30 min long. All extractions were performed in duplicate. The following extracts were thus obtained: total extract (**RTE**), spent plant material extract (**RSE**), SC-CO_2_ (100 bar, 40 °C) spent extract (**RSC1**), SC-CO_2_ (100 bar, 50 °C) spent extract (**RSC2**), SC-CO_2_ (100 bar, 60 °C) spent extract (**RSC3**). The extract yields are provided in Table 1.

### 2.4. Phytochemical Characterization

#### 2.4.1. GC-MS Analysis

A TRACE gas chromatograph [18] with an ISQ™ mass spectrometer (MS) from Thermo Fisher (Waltham, MA, USA) was used. The chromatographic separations were conducted on a Zebron™ ZB-5MS (30 m × 0.25 mm i.d., 0.25 µm film thickness) from Phenomenex (Torrance, CA, USA). Helium at a flow rate of 1.43 mL/min was the carrier gas. The inlet temperature was 250 °C, the split ratio was 50:1, and the injection volume was 1 μL. The oven temperature was held for 4 min at 60 °C; then it was increased to 280 °C at a rate of 10 °C/min and held for 5 min; finally, it was ramped to 300 °C at a rate of 10 °C/min and held for 10 min. The following MS settings were used: *m*/*z*: 50 to 350 amu; ionization energy: 70 eV; transfer line temperature: 320 °C; and source temperature: 230 °C. The linear retention indices were determined for each peak using a C8–C20 standard mixture of *n*-alkanes and compared with those of the literature. Furthermore, the recorded mass spectral information was compared with that from the NIST11 database. All measurements were performed in triplicate.

#### 2.4.2. LC-HRMS/MS Analysis

An Agilent 1200 HPLC (Agilent Technologies, Palo Alto, CA, USA) with an accurate-mass quadrupole time-of-flight MS detector (G6530B) was used. The chromatographic separations were conducted on a Gemini C18 column (100 mm × 2 mm i.d., 3 μm) from Phenomenex (Torrance, CA, USA). The mobile phases comprised water (A) and acetonitrile (B), both acidified with 0.1% formic acid. The run started with 10% B and linearly increased to 60% B in 45 min at a flow rate of 0.2 mL/min; the injection volume was 10 μL. The following MS settings were used: *m*/*z:* 100–1700 amu; negative ionization mode; carrier gas (nitrogen) flow rate and temperature of 10 L/min and 275 °C, respectively; sheath gas (nitrogen) flow rate and temperature of 12 L/min and 325 °C, respectively; nebulizer pressure: 35 psi; capillary voltage: 4000 V; nozzle voltage: 1000 V; skimmer: 65 V; fragmentor: 140 V; and collision-induced dissociation: 30 V. The recorded mass spectral information was compared with that from databases and the literature.

#### 2.4.3. Total Phenolic and Flavonoid Content

The total phenolic content (TPC) and total flavonoid content (TFC) were determined spectrophotometrically as described in [19]. Data were expressed as mg gallic acid equivalents (GAE)/g extract in TPC and mg rutin equivalents (RE)/g extract in TFC. All measurements were performed in triplicate.

### 2.5. Biological Activity Evaluation

#### 2.5.1. Antimicrobial Assays

The microdilution method was used to determine the antimicrobial activity according to the European Committee on Antimicrobial Susceptibility Testing [20]. MH broth and MH broth with 2% glucose were used to grow the bacteria and yeasts, respectively. The following microbial strains were tested: *Bacillus subtilis* ATCC 6633, *Candida albicans* ATCC 2091, *Candida glabrata* ATCC 90030, *Candida parapsilosis* ATCC 22019, *Enterococcus faecalis* ATCC 29212, *Escherichia coli* ATCC 25922, *Klebsiella pneumoniae* ATCC 13883, *Micrococcus luteus* ATCC 10240, *Proteus mirabilis* ATCC 12453, *Pseudomonas aeruginosa* ATCC 90271, *Salmonella* Typhimurium ATCC 14028, *Staphylococcus aureus* ATCC 25923, *Staphylococcus aureus* ATCC BAA-1707 (methicillin-resistant strain), and *Staphylococcus epidermidis* ATCC 12228. The minimum inhibitory concentration (MIC) was calculated and reported for each sample and strain. All experiments were performed in triplicate.

#### 2.5.2. Antioxidant Assays

The DPPH radical scavenging, ABTS radical scavenging, cupric ion reducing antioxidant capacity (CUPRAC), ferric ion reducing antioxidant power (FRAP), metal chelating activity (MCA), and phosphomolybdenum (PBD) were determined as presented in [19,21]. The activity data were expressed as mg Trolox equivalents (TE)/g extract in DPPH, ABTS, CUPRAC, and FRAP assays; mg EDTA equivalents (EDTAE)/g extract in the MCA assay; and mmol TE/g extract in the PBD assay.

#### 2.5.3. Enzyme-Inhibitory Assays

AChE, BChE, tyrosinase, amylase, and glucosidase inhibition were determined as presented in [19,21]. The activity data were expressed as mg galanthamine equivalents (GALAE)/g extract in the AChE and BChE assays, mg kojic acid equivalents (KAE)/g extract in tyrosinase assay, and mmol acarbose equivalents (ACAE)/g extract in amylase and glucosidase assays.

### 2.6. Statistical and Data Processing

Data are presented as mean ± standard deviation of the respective number of replicates. One-way analysis of variance with Tukey’s post-hoc test was conducted; *p* < 0.05 was considered statistically significant. The relationship between rosemary compounds vs. antimicrobial, antioxidant, and enzyme-inhibitory activities was assessed by calculating the Pearson correlation coefficient. Principal component analysis (PCA) and clustered image maps (CIM) were also performed, with the phytochemical data log transformed. The statistical analysis was done using R software v. 4.1.2 (R Foundation for Statistical Computing, Vienna, Austria).

## 3. Results and Discussion

### 3.1. GC-MS Characterization of Essential Oil and SC-CO_2_ Rosemary Extracts

In this section, rosemary extracts rich in volatile compounds were obtained by hydrodistillation and SC-CO_2_ extraction and characterized by GC-MS. The operating SC-CO_2_ pressure (100 bar) and temperature range (40–60 °C) were selected based on previous systematic studies that presented a high recovery of rosemary volatiles under these conditions [11,15]. The EO yield was significantly higher than the SC-CO_2_ yields (Table 1). This can be related to the different extraction mechanisms between the hydrodistillation and SC-CO_2_ extraction. Hydrodistillation allows the recovery of only high-vapor-pressure (volatile, ‘distillable’) compounds, whereas SC-CO_2_ extraction allows the recovery of compounds mostly based on their polarity and to a lower extent based on their vapor pressure. The high lipophilicity of the solvent (SC-CO_2_) would allow high extraction rates of lipophilic compounds, including the low-polarity terpenes.

Within the three SC-CO_2_ extracts, the yield decreased with the increase in temperature. This is in connection with the fact that temperature increments are known to reduce solvent density and negatively impact the solubility and extractability of compounds [22]. According to the GC-MS profiling (Table 2), the rosemary EO (**REO**) was characterized by 46 compounds, primarily monoterpenes (~97.2% of the total GC-MS peak area). The putative identity of the volatile compounds was established by comparing the linear retention indices with those of the literature data and the recorded mass spectra with those of NIST11 database. However, due to the lack of authentic standards, only a partial structural identification is possible with these resources.

**Figure 1:**
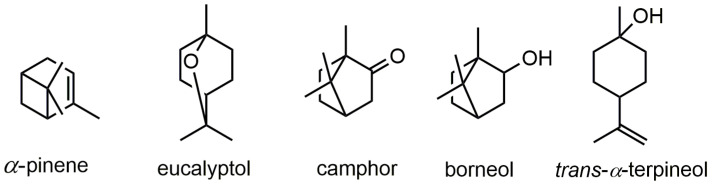
Proposed chemical structures of the main volatile terpenes identified in rosemary essential oil.

The major volatile compounds (Figure 1) were represented by eucalyptol (41.7%), camphor (13.5%), and *α*-pinene (13.27%). Similarly, Ramzi et al. [23] documented eucalyptol (29.31%), camphor (24.7%), and *α*-pinene (12.8%) as the dominant terpenes in rosemary EO. In addition, Ouknin et al. [24] reported eucalyptol (27.6%), *α*-pinene (26.6%), verbenone (5.3%), camphene (4.5%), and camphor (4.3%) as the main compounds of rosemary EO.

Concerning the three SC-CO_2_ extracts, significant differences in the profiles of the volatile compounds were noticed. The SC-CO_2_ extraction at 100 bar and 40 °C (**RC1**) recovered the lowest number (17 compounds) and amount of terpenes (only 41.7% of the total GC-MS peak area). Eucalyptol remained the predominant compound in this extract, but its level was considerably low (13.8%). However, *α*-pinene (1.1%) and camphor (0.3%) were found in traces. The extract obtained at 100 bar and 50 °C (**RC2**) displayed a significantly high number (48 compounds) and concentration of volatile compounds (95.6% of the total GC-MS peak area). Eucalyptol (29.8%) and camphor (18.3%) were the major terpenes in **RC2**; in addition, the levels of trans-*α*-terpineol (9.5%) and caryophyllene (9.8%) were dramatically increased as compared to those of **REO** and **RC1**. Lastly, the third SC-CO_2_ extraction conditions (100 bar and 60 °C) allowed the recovery of 24 terpenes (accounting for 98.6% of the total GC-MS peak area). Nonetheless, the concentration of eucalyptol decreased to 2.71%, whereas the concentration of camphor was kept high (at 18.8%). Furthermore, borneol (10.4%), trans-*α*-terpineol (20.4%), and caryophyllene (21.5%) reached their highest values in **RC3**.

Overall, it can be noticed that hydrodistillation was clearly more selective in recovering rosemary terpenes. However, the selectivity of the SC-CO_2_ extraction toward volatiles increased considerably by increasing the temperature from 40 to 60 °C. Most likely, the SC-CO_2_ process, especially at 100 bar and 40 °C, allowed the simultaneous extraction of non-volatile lipophilic compounds (e.g., waxes, fatty acids, lipophilic pigments, etc.). It is also worth emphasizing that the ratio of monoterpenes/sesquiterpenes significantly decreased from 36/1 in the **REO** to 4/1, 4.4/1, and 1.7/1 in **RC1**, **RC2,** and **RC3**, respectively. Even though various SC-CO_2_ extraction conditions allow the efficient recovery of terpenes, the extraction yields, qualitative profile, and quantitative data of terpenes are significantly altered compared to those with hydrodistillation. Similar conclusions were also reported in previous works [12].

### 3.2. LC-HRMS/MS Characterization of the Total, Post-Distillation, and Post-SC-CO_2_ Rosemary Extracts

In this section, various rosemary extracts, namely total (unspent plant material, **RTE**), post-hydrodistillation residual water (**RWE**), post-hydrodistillation spent material (**RSE**), and post-SC-CO_2_ spent material (**RSC1**, **RSC2**, and **RSC3**) extracts, were obtained and characterized by LC-HRMS/MS. **RWE** was characterized by the highest extraction yield (25.6%); interestingly, the yield of **RSE** was significantly low (9.2%) (Table 1). The post-SC-CO_2_ materials allowed extraction yields between 17.1% and 20.9%. The order is correlated with the SC-CO_2_ extraction yields: the higher the SC-CO_2_ extraction yields, the lower the post-SC-CO_2_ extraction yields (Table 1). The LC-HRMS/MS profiling (Table 3) allowed the annotation of 25 specialized metabolites belonging to various phytochemical classes. The putative identity of the compounds was established by comparing the spectro-chromatographic data with those presented in the literature [2,25,26,27,28] and relevant databases (METLIN, KNApSacK, PubChem, NIST Chemistry WebBook). However, due to the lack of authentic standards, only a partial structural identification was possible with these resources.

Danshensu (**2**), hydroxybenzoic acid (**3**), caffeic acid (**4**), rosmarinic acid (**6**), and salvianolic acid A (**8**) were found as typical phenolic acids in the analyzed samples. In addition, seven flavonoids were spotted. They were putatively labeled as free aglycones: gallocatechin (**5**), cirsimaritin (**11**), ladanein (**12**), genkwanin (**16**), glycosylated flavonoids: luteolin-*O*-glucuronide (**7**), and two isomeric luteolin-*O*-acetylglucuronides (**9** and **10**). Besides ladanein, the other phenolic acids and flavonoids were previously documented in rosemary extracts [2,27,28]. Diterpenes constituted the representative class of phytochemicals (12 compounds). A few diterpenes were derivatives of rosmanol (**13**), such as epirosmanol (**14**), isorosmanol (**15**), epiisorosmanol (**17**), epirosmanol methyl ether (**18**), and methoxyrosmanol (**19**). The remaining diterpenes were either derivatives of carnosol (**20**), namely carnosic acid (**23** and **25**) and carnosic acid methyl ester (**24**), or two isomers of rosmadial (**21** and **22**). Previously, rosemary extracts were shown to be abundant in similar diterpenic compounds [2,27,28].

The by-product extracts that resulted after the hydrodistillation and SC-CO_2_ extraction of rosemary can be regarded as rich sources of phytochemicals, especially phenolic compounds, such as phenolic acids, flavonoids, and diterpenes. Compared to the total (unspent material), no substantial qualitative differences were spotted in the spent material extracts. **RTE** and the three post-SC-CO_2_ extracts showed a very similar metabolite profile. In the **RWE,** several non-polar diterpenes (e.g., **17**, **18**, **19**, **21**, **22**, and **25**) were not present, which could be linked to the high polarity of the solvent (water). However, **RSE** showed the highest abundance of compounds. Several hypotheses can be formulated. For instance, constituents found in small amounts in the original (unspent) plant materials could become more accessible to the solvent extraction that follows hydrodistillation. On the other hand, due to the long exposure of the plant material to boiling water, a cell permeation effect can be assumed, favoring the subsequent extraction of the metabolites. In addition, the harsh hydrodistillation conditions (high temperatures and long exposure times) can also lead to the formation of phenolic artifacts in the spent extracts.

**Figure 2:**
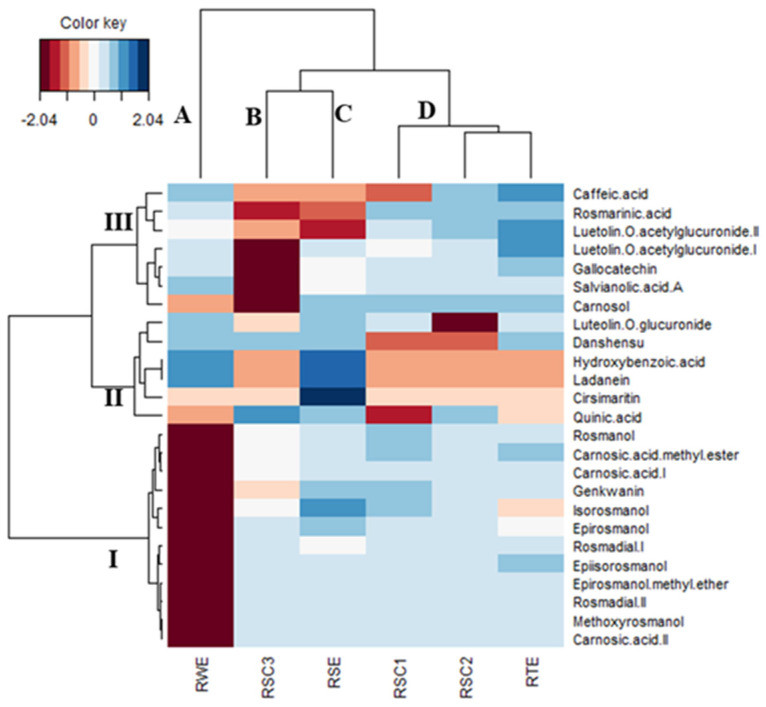
Global overview of the phytochemical differences among total, post-distillation, and post-SC-CO_2_ rosemary extracts (Red color: low content. Blue color: high content).

To find more significant differences in the six extracts, a CIM analysis was next performed with the logarithmically transformed and scaled semi-quantitative data (peak area extracted from the base peak chromatograms of the LC-HRMS/MS analyses). As shown in Figure 2, the samples were distinguishable from each other, even if they seemed to form four clusters. Moreover, to describe the compounds characterizing each cluster, three blocks (I-III) were defined. In brief, **RWE** (cluster A) and **RSC3** (cluster B) contained low concentrations of compounds grouped in blocks I and III (Figure 2). Cirsimaritin, ladanein, and hydroxybenzoic acid were abundant in cluster C comprising **RSE**. In contrast, the samples of cluster D (**RSC1**, **RSC2**, and **RTE**) had low levels of the compounds mentioned above. In this cluster, **RTE** contained the highest concentration of caffeic acid and luteolin-*O*-acetylglucuronides.

### 3.3. Total Phenolic and Flavonoid Content of the Total, Post-Distillation, and Post-SC-CO_2_ Rosemary Extracts

In this section, the TPC and TFC of the total (**RTE**), post-distillation (**RSE**, **RWE**), and post-SC-CO_2_ (**RSC1-3**) rosemary extracts were determined. As can be seen from Table 4, the highest TPC was detected in **RWE** (108.10 mg GAE/g), followed by **RTE** (99.36 mg GAE/g), **RSC2** (98.02 mg GAE/g), and **RSC1** (97.68 mg GAE/g). **RSE** contained the lowest TPC. Regarding TFC, the highest content was recorded in **RSC2** (32.58 mg RE/g) and the lowest in **RSE** (19.86 mg RE/g). Altogether, **RWE** can be regarded as an extract with a very high amount of both phenolic and flavonoid compounds. Different results regarding the total bioactive content of rosemary extracts have been reported in the literature [29,30]. In a recent paper by Zeroual et al. [31], the TPC and TFC in rosemary extracts were dependent on extraction methods (Soxhlet and maceration) and solvents (hexane, ethyl acetate, methanol, and ethanol). In their study, the highest TPC (34.98 mg GAE/g) was lower than that of the current findings. In another study [32], sixty Jordanian plants were investigated, with the highest TPC recorded in the rosemary extract (101.339 mg GAE/g).

### 3.4. Post-Distillation and Post-SC-CO_2_ Rosemary Extracts as Antimicrobials

The extensive use of antibiotics and the rapid emergence of multi-drug-resistant microbial strains represent severe issues for modern medicine. Numerous approaches are currently under evaluation, such as using novel plant-based antimicrobials with superior efficiency and safety profiles [33]. Various studies have repeatedly brought to attention the antimicrobial activity of rosemary EO and solvent extracts [34,35,36,37,38,39]. Thus, in this section, the activity of the ten rosemary raw and by-product extracts was evaluated by the micro-dilution method in a panel of 14 pathogenic strains. The criteria proposed by Kuete and Efferth [40] were used to categorize the observed activity into significant (MIC < 100 mg/L) and moderate-to-weak (MIC > 100 mg/L) activity. According to the results presented in Table 5, it was observed that **REO**, **RC2**, **RC3**, and **RWE** showed practically no relevant antimicrobial activity (MIC > 250 mg/L). In connection with the phytochemical composition (Table 2 and Table 3), it can be assumed that the rosemary extracts rich in lipophilic compounds (the case of **REO**, **RC2,** and **RC3**) or hydrophilic compounds (the case of **RWE**) were inactive. Generally, the Gram-negative bacteria and yeasts were not inhibited by any extract. The most sensitive strains (MIC = 15.6 mg/L) were *S. aureus* after the treatment with **RSE** and **RSC1** and *M. luteus* after the treatment with **RC1**. With MIC values of 31.3 mg/L, **RC1**, **RTE**, **RSC2**, and **RSC3** also potently inhibited *S. aureus*. A similar effect was exhibited by **RC1** against *S. epidermidis*, **RSE** against *M. luteus* and *E. faecalis*, and **RSC1** against *M. luteus*. *S. aureus* MRSA was sensitive (MIC = 62.5 mg/L) to **RC1** and **RSE**, whereas *B. cereus* was inhibited to the same extent by **RC1**, **RTE**, and **RSE**.

**Figure 3:**
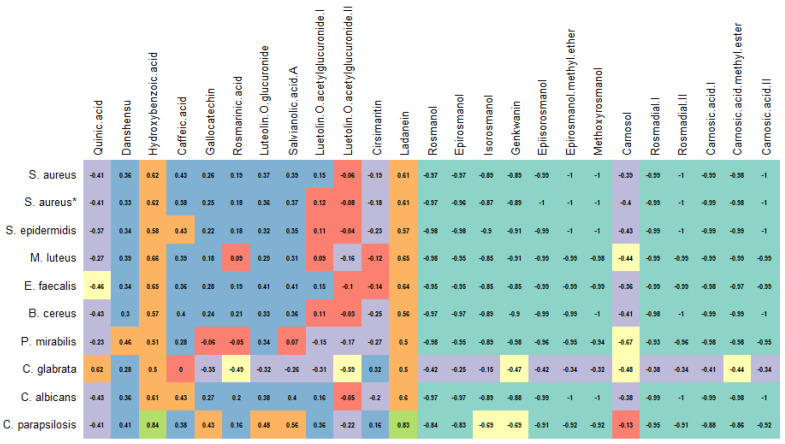
Correlation between polyphenols and antimicrobial activities of rosemary extracts.

**Figure 4:**
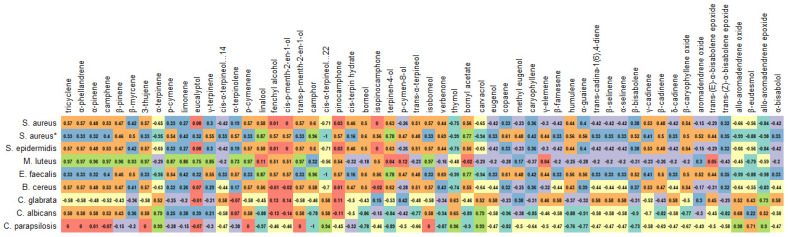
Correlation between volatile metabolites and antimicrobial activities of rosemary extracts.

The high MIC values for the rosemary EO agree with those of the literature [36,37]. For example, Hussain et al. [38] reported MIC values ranging from 300 mg/L to 1720 mg/L for rosemary EO against various Gram-positive and Gram-negative bacteria, whereas Ojeda-Sana et al. [39] documented values of 1000–2500 mg/L against *S. aureus*, *E. faecalis*, *E. coli*, and *K. pneumonia*. In contrast, various solvent extracts were more potent as antimicrobial agents. Amaral et al. [34] reported MIC values ranging from 16 to 256 mg/L for rosemary extracts obtained with ethyl acetate, dichloromethane, and ethanol against *S. aureus*, *S. epidermidis*, and *B. cereus*. Karadag et al. [35] showed MIC values between 78 and 156 mg/L against *S. aureus*, *E. faecalis*, and *H. pylori* for a hexane rosemary extract. In summary, it can be stated that the post-distillation and post-SC-CO_2_ extracts are more efficient antimicrobial agents than the EO and SC-CO_2_ extracts. In addition, some polyphenolic compounds’ (e.g., epiirosmanol with *S. aureus* and *C. albicans*) volatile metabolites (e.g., camphor with *C. parapsilosis*) seemed to have been correlated to some extent with the antimicrobial activity (Figure 3 and Figure 4).

### 3.5. Post-Distillation and Post-SC-CO_2_ Rosemary Extracts as Antioxidants

Over the past decade, antioxidants have become increasingly popular in the treatment of oxidative-stress-related diseases, such as cardiovascular disease, diabetes, and cancer [41]. Thus, intensive efforts are carried out to identify new and safer sources of antioxidants. In this section, the antioxidant properties of rosemary extracts obtained from raw and by-product materials were investigated in six complementary assays, including radical quenching (ABTS and DPPH), reducing power (CUPRAC and FRAP), phosphomolybdenum, and metal chelating. The results are presented in Table 6. In the radical scavenging assays, the best ability was noted in **RSC2** (DPPH: 173.49 mg TE/g; ABTS: 202.19 mg TE/g), followed by **RWE** (DPPH: 164.08 mg TE/g; ABTS: 179.76 mg TE/g), and **RTE** (DPPH: 144.17 mg TE/g; ABTS: 155.03 mg TE/g). The weakest abilities for both radical quenching abilities were found in the samples obtained from the supercritical CO_2_ extraction, and they can be ranked as **RC1** > **RC2** > **RC3**. The observed radical scavenging abilities were adversely affected by the increase in temperature during the supercritical CO_2_ extraction procedure. In the spent extracts from the SC-CO_2_ extractions, the radical scavenging ability decreased in the order **RSC2** > **RSC1** > **RSC3**, which corresponds to the level of total bioactive compounds. Additionally, when **REO** was compared to the SC-CO_2_ extracts, **REO** demonstrated a higher radical scavenging ability than **RC3**. With values of 396.28 mg TE/g in CUPRAC and 205.38 mg TE/g in FRAP, **RWE** can be an excellent reducing agent compared to other samples. The reduction power of SC-CO_2_ and post-SC-CO_2_ extracts followed the same pattern as the radical scavenging activity. From these findings, it could be concluded that similar compounds could play a key role in the assays. As can be seen in Figure 3, some compounds (e.g., rosmarinic acid, luteolin, and caffeic acid) correlated strongly with radical scavenging and reducing abilities. Consistent with our approach, several researchers have already described these compounds as powerful antioxidants [18,42,43]. In addition, some volatile metabolites, such as thymol and carvacrol, could contribute significantly to the observed radical scavenging and reducing activities of **REO** and SC-CO_2_ extracts.

**Figure 5:**
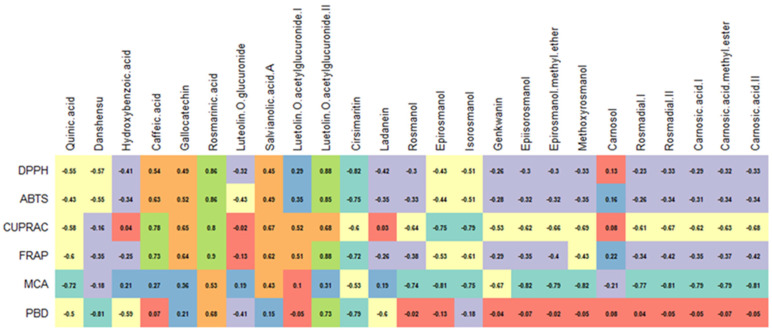
Correlation between polyphenols and antioxidant activities of rosemary extracts.

**Figure 6:**
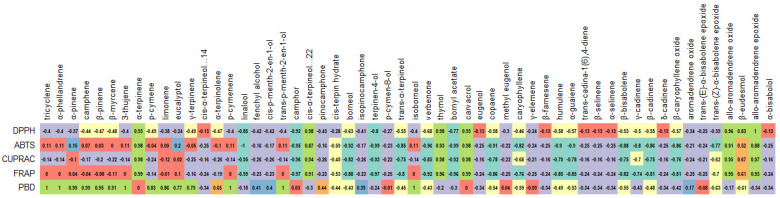
Correlation between volatile metabolites and antioxidant activities of rosemary extracts.

The highest metal chelating ability was observed in **RWE** with 8.63 mg EDTAE/g, followed by **RSC1** (5.34 mg EDTAE/g) and **RSC2** (3.13 mg EDTAE/g). Surprisingly, all non-polar samples, namely **REO** and SC-CO_2_ extracts, showed no chelating effects. From Figure 5, only two compounds (rosmarinic acid and salvianolic acid A) moderately correlated with the chelating activity. In this sense, the observed ability can be explained by the presence of non-phenolic chelators, such as peptides or polysaccharides. In contrast to other assays, the highest value in the phosphomolybdenum assay was achieved by **REO** (18.08 mmol TE/g), followed by **RC1**, **RC2**, and **RC3**. The non-polar samples were more active than the polar samples. This fact was also observed in the correlation analysis. As shown in Figure 6, numerous volatile compounds were strongly associated with this propensity. These results are consistent with those of the literature that reported potent phosphomolybdenum properties for EOs [44,45]. Additionally, significant antioxidant properties of rosemary extracts, post-distillation, or essential oils have been reported in several studies [11,46,47].

### 3.6. Post-Distillation and Post-SC-CO_2_ Rosemary Extracts as Enzyme Inhibitors

**Figure 7:**
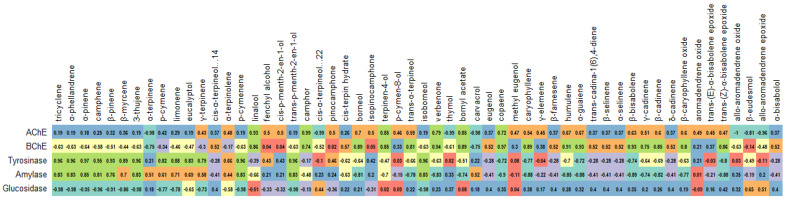
Correlation between volatile metabolites and enzyme-inhibitory activities of rosemary extracts.

In this section, the inhibitory effects of rosemary raw and by-product extracts against AChE, BChE, tyrosinase, amylase, and glucosidase were investigated (Table 7). In the AChE inhibition, the best result was achieved by **RSC3** with 3.80 mg GALAE/g. However, its ability was similar to that of **RSC1**, **RC3,** and **RC2**. Interestingly, none of the polar extracts were active on BChE except **RSC3**. In contrast to the AChE inhibition, **RC3** exhibited the most potent BChE inhibitory effect (3.01 mg GALAE/g). As shown in Figure 7, specific terpenoids, including linalool, terpinene-4-ol, and camphor, may be responsible for the anti-cholinesterase properties observed in the EO and SC-CO_2_ extracts. In this sense, a good agreement with previous studies was found [48,49,50]. In addition, the cholinesterase-inhibiting effects of rosemary have been reported in several studies.

The highest tyrosinase inhibition was provided by **REO** with 59.23 mg KAE/g, followed by **RC1**, **RC2**, and **RSC1**. The anti-tyrosinase activity of post-SC-CO_2_ extracts was less potent than their supercritical counterparts. This fact could be explained by some volatile compounds (*α*-pinene, *β*-pinene, *p*-cymene, etc.) and was confirmed as shown in Figure 7. The residual water, spent, and total extracts showed similar anti-tyrosinase abilities.

**Figure 8:**
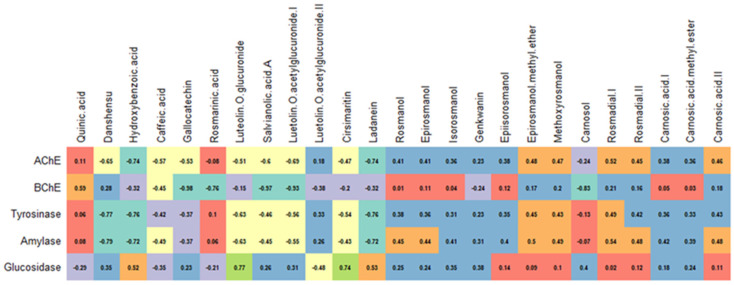
Correlation between polyphenols and enzyme-inhibitory activities of rosemary extracts.

**REO** achieved the most substantial inhibition value (0.39 mmol ACAE/g) in the case of amylase. Surprisingly, the same sample was not active on glucosidase; the highest glucosidase inhibitory activity was found in **RSE** (1.24 mmol ACAE/g), but the value was similar to **RC1** (1.20 mmol ACAE/g) and **RC2** (1.17 mmol ACAE/g). These results suggest terpenoids, such as *α*-pinene, *β*-pinene, or *α*-phellandrene, might be attributed to amylase inhibition. At the same time, some phenolics (including luteolin and ladanein) might also be the main players in the glucosidase-inhibitory capacity (Figure 8). Moreover, the mentioned compounds have been reported to have an inhibitory effect on the enzymes [51,52,53].

### 3.7. Multivariate Analysis

The application of multivariate tools in biochemical sciences has been proven to be extremely convenient since it enables the cluster of different biological activities of different samples [54]. To establish the global overview of the similarities and differences between all the rosemary samples in terms of their bioactivities, two multivariate methods (PCA, CIM) were applied. Before the PCA analysis, the data were scaled to ensure the equal influence of all the bioactivities analyzed. In the CIM analysis, clusters were formed by the Ward method, and Euclidean distance was applied as a measure of diversity in the cluster analysis.

**Figure 9:**
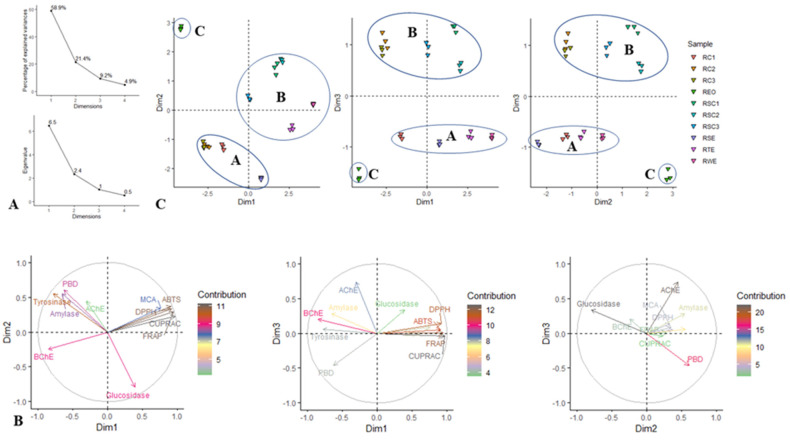
Principal component analysis. (**A**) Percentage of explained variance and eigenvalue. (**B**) Correlation circle showing the relationship of biological activities on each dimension of PCA. (**C**) Scatter plot showing the distribution of the samples in the factorial plan derived from the three retained dimensions.

In Figure 9A, the first three dimensions represented practically 90% of the variance. The relationship of the three dimensions with the bioactivities is presented in Figure 9B. Due to its high percentage of explained variance (58.9%), the first dimension was linked to several bioactivities compared to the other two dimensions. Indeed, dimension 1 was positively correlated with DPPH, ABTS, CUPRAC, and FRAP and negatively correlated with BChE. The second dimension, which accounted for 21.4% of the variance, was positively bound to phosphomolybdenum, amylase, and tyrosinase and negatively bound to glucosidase. In the third dimension, only AChE showed a significant correlation.

**Figure 10:**
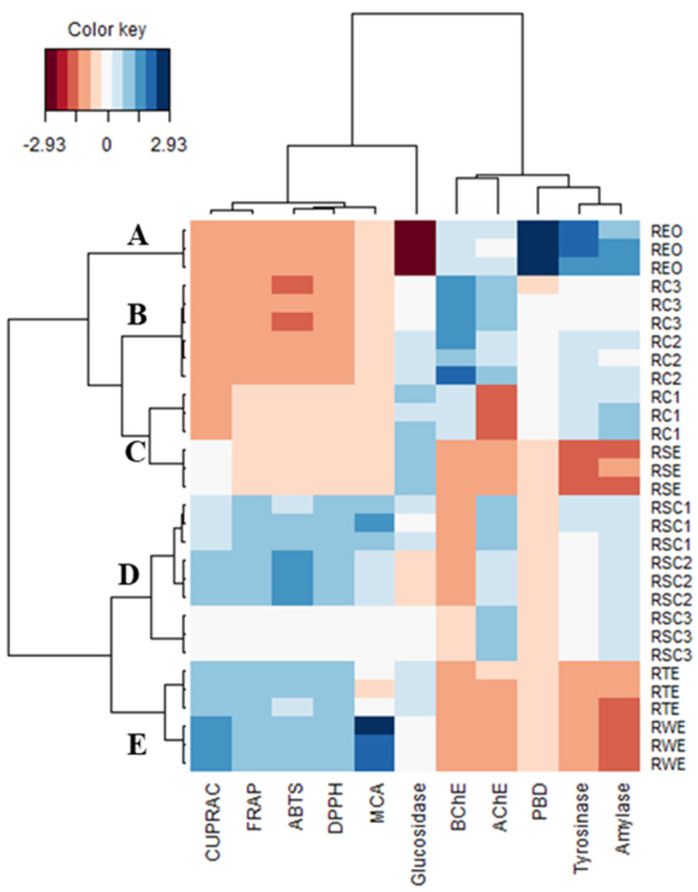
Clustered image map analysis on biological activities dataset. (Red color: low bioactivity. Blue color: high bioactivity).

Figure 9C shows the division of the tested samples based on the three dimensions. In each scatter plot, the samples were divided into three clusters. In each of these plots, the **REO** sample formed a standalone cluster. In addition, the samples forming clusters A and B in the first scatter plot differed from those constituting the same clusters in the remaining scatter plot. Thereby to evaluate the accuracy of the PCA classification, the clustering was adequately identified by the CIM analysis. Five clusters grouped into two large clusters were obtained (Figure 10). Cluster A comprised **REO**, which had remarkable phosphomolybdenum, anti-tyrosinase, and anti-amylase activities. Cluster B included **RC3** and **RC2**, which demonstrated a relatively high anti-BChE activity. Cluster C contained **RC1** and **RSE**. Clusters D (**RSC1**, **RSC2**, and **RSC3**) and E (**RTE** and **RWE**) were distinguished from the other clusters by their antioxidant activity. In short, the terpene-containing extracts (**REO, RC1-RC3**) showed better anti-enzymatic activity, while the remaining extracts showed good antioxidant activity.

## 4. Conclusions

In this work, rosemary extracts obtained from raw materials (essential oil, SC-CO_2_, and total extracts) and post-distillation and post-SC-CO_2_ materials were comparatively assessed for the first time from a phytochemical (GC-MS, LC-HRMS/MS) and multi-biological (antimicrobial, antioxidant, enzyme-inhibitory) approach. Overall, it can be concluded that the by-products can find uses beyond those of the terpene-rich extracts (EO, SC-CO_2_) that are conventionally used as food preservatives (antioxidants) or aroma-active ingredients. The antimicrobial, antioxidant, and enzyme-inhibitory results could provide initial evidence for the health-promoting effects of the post-distillation and post-SC-CO_2_ samples, which can constitute novel materials for the pharmaceutical, cosmetic, and nutraceutical industries. Furthermore, this can lead to finding new ways of exploiting the large amounts of waste produced worldwide by the rosemary essential oil industry, with beneficial environmental, technological, and economic advantages.

## Figures and Tables

**Table 1:** Extraction yields of rosemary extracts.

Extract	Code	Yield [g/100 g]
*Extracts from raw materials*		
Essential oil	REO	2.5 ± 0.1 *
SC-CO_2_ (100 bar, 40 °C) extract	RC1	1.5 ± 0.2
SC-CO_2_ (100 bar, 50 °C) extract	RC2	0.8 ± 0.2
SC-CO_2_ (100 bar, 60 °C) extract	RC3	0.2 ± 0.0
Total extract	RTE	16.8 ± 3.1
*Extracts from post-distillation materials*		
Distillation (residual) water extract	RWE	25.6 ± 1.1
Spent plant material extract	RSE	9.2 ± 0.8
*Extracts from post-SC-CO_2_ materials*		
SC-CO_2_ (100 bar, 40 °C) spent extract	RSC1	17.1 ± 2.1
SC-CO_2_ (100 bar, 50 °C) spent extract	RSC2	18.6 ± 0.8
SC-CO_2_ (100 bar, 60 °C) spent extract	RSC3	20.9 ± 1.8

* mL oil/100 g plant material.

**Table 2:** GC-MS profile (tentative annotation) of the essential oils obtained from thyme, oregano, and basil.

No.	Compound	LRI ^a^	REO	RC1 (100 bar, 40 °C)	RC2 (100 bar, 50 °C)	RC3 (100 bar, 60 °C)
			% ^b^			
1	Tricyclene	924	0.10 ± 0.01	–	–	–
2	*α*-Phellandrene	927	0.02 ± 0.00	–	–	–
3	*α*-Pinene	935	**13.27 ± 0.14**	1.07 ± 0.08	1.91 ± 0.07	–
4	Camphene	952	3.88 ± 0.03	–	0.55 ± 0.02	–
5	*β*-Pinene	980	2.59 ± 0.02	–	0.76 ± 0.02	–
6	*β*-Myrcene *	989	1.18 ± 0.01	–	0.48 ± 0.01	–
7	3-Thujene	1007	0.15 ± 0.00	–	–	–
8	*α*-Terpinene	1018	0.64 ± 0.00	1.85 ± 0.08	0.26 ± 0.01	–
9	*p*-Cymene	1027	2.72 ± 0.01	–	1.58 ± 0.03	–
10	Limonene *	1031	2.67 ± 0.04	0.36 ± 0.02	1.46 ± 0.03	–
11	Eucalyptol	1034	**41.68 ± 0.42**	**13.81 ± 0.89**	**29.75 ± 1.14**	2.71 ± 0.01
12	*γ*-Terpinene	1060	0.47 ± 0.01	–	0.30 ± 0.01	–
13	*cis*-*α*-Terpineol	1072		–	0.17 ± 0.01	–
14	*α*-Terpinolene	1087	0.20 ± 0.00	–	0.17 ± 0.01	–
15	*p*-Cymenene	1091	0.06 ± 0.01	–	–	–
16	Linalool	1099	1.38 ± 0.03	–	2.32 ± 0.01	2.94 ± 0.05
17	Fenchyl alcohol	1121	0.07 ± 0.01	–	0.10 ± 0.01	–
18	*cis*-*p*-Menth-2-en-1-ol	1127	0.04 ± 0.00	–	0.05 ± 0.01	–
19	*trans*-*p*-Menth-2-en-1-ol	1145	0.04 ± 0.00	–	–	–
20	Camphor	1150	**13.52 ± 0.06**	0.27 ± 0.04	**18.33 ± 0.27**	**18.75 ± 0.22**
21	*cis*-*α*-Terpineol	1159	0.04 ± 0.00	5.54 ± 0.38	0.03 ± 0.00	–
22	Pinocamphone	1163	0.17 ± 0.03	–	0.21 ± 0.00	–
23	*cis*-Terpin hydrate	1173	–	0.46 ± 0.03	–	2.57 ± 0.06
24	Borneol	1176	**4.47 ± 0.05**	3.54 ± 0.19	**6.74 ± 0.01**	**10.40 ± 0.16**
25	Isopinocamphone	1179	0.05 ± 0.01	–	0.06 ± 0.02	–
26	Terpinen-4-ol	1183	1.05 ± 0.03	0.41 ± 0.03	1.45 ± 0.02	2.18 ± 0.02
27	*p*-Cymen-8-ol	1188	0.03 ± 0.00	–	0.09 ± 0.01	–
28	*trans*-*α*-Terpineol	1197	**5.89 ± 0.09**	**5.13 ± 0.33**	**9.52 ± 0.04**	**20.41 ± 0.23**
29	Isoborneol	1198	0.03 ± 0.00	–		–
30	Verbenone	1202	0.20 ± 0.01	–	0.68 ± 0.01	1.01 ± 0.05
31	Thymol	1281	0.09 ± 0.01	0.55 ± 0.05	0.08 ± 0.01	–
32	Bornyl acetate	1286	0.31 ± 0.01		0.61 ± 0.02	0.96 ± 0.02
33	Carvacrol	1293	0.12 ± 0.04	0.33 ± 0.02	0.05 ± 0.01	–
34	Eugenol	1350	–	–	0.08 ± 0.01	–
35	Copaene	1380	0.05 ± 0.00	–	0.48 ± 0.03	0.70 ± 0.05
36	Methyl eugenol	1398	0.04 ± 0.01	–	0.12 ± 0.01	–
37	*β*-Caryophyllene *	1428	1.43 ± 0.06	3.24 ± 0.18	**9.80 ± 0.36**	**21.47 ± 0.37**
38	*γ*-Elemene	1447	0.04 ± 0.00	–	0.17 ± 0.02	–
39	*β*-Farnesene	1453	–	–	0.02 ± 0.00	–
40	*α*-Humulene *	1464	0.20 ± 0.01	–	1.33 ± 0.07	2.77 ± 0.03
41	*α*-Huaiene	1481	0.04 ± 0.01	–	0.43 ± 0.04	0.81 ± 0.05
42	*trans*-Cadina-1(6),4-diene	1484	–	–	0.09 ± 0.02	–
43	*β*-Selinene	1498	–	–	0.14 ± 0.01	–
44	*α*-Selinene	1504	–	–	0.22 ± 0.02	–
45	*β*-Bisabolene	1511	–	–	0.36 ± 0.03	0.74 ± 0.01
46	*γ*-Cadinene	1520	0.02 ± 0.00	–	0.19 ± 0.01	0.65 ± 0.02
47	*β*-Cadinene	1524	0.09 ± 0.01	–	0.88 ± 0.05	2.15 ± 0.04
48	*δ*-Cadinene	1528	–	–	0.23 ± 0.03	–
49	*β*-Caryophyllene oxide *	1593	0.26 ± 0.02	–	0.91 ± 0.06	3.26 ± 0.05
50	Aromadendrene oxide	1603	0.04 ± 0.01	–	0.07 ± 0.01	–
51	*trans*-(*E*)-*α*-Bisabolene epoxide	1620	0.04 ± 0.01	–	0.17 ± 0.02	–
52	*trans*-(*Z*)-*α*-Bisabolene epoxide	1644	0.06 ± 0.01	0.46 ± 0.00	1.05 ± 0.08	3.12 ± 0.13
53	*allo*-Aromadendrene oxide	1647	0.07 ± 0.01	0.53 ± 0.01	–	–
54	*β*-Eudesmol	1663	0.25 ± 0.02	2.56 ± 0.12	0.62 ± 0.06	1.20 ± 0.06
55	*allo*-Aromadendrene epoxide	1678	0.11 ± 0.01	1.58 ± 0.06	0.37 ± 0.05	–
56	*α*-Bisabolol *	1690	–	–	0.03 ± 0.01	–
	*Hydrocarbon monoterpenes*		*27.96 ± 0.14*	*3.56 ± 0.10*	*7.30 ± 0.18*	*–*
	*Oxygenated monoterpenes*		*69.23 ± 0.06*	*29.78 ± 1.88*	*70.62 ± 1.24*	*61.93 ± 0.65*
	*Hydrocarbon sesquiterpenes*		*1.86 ± 0.09*	*3.24 ± 0.18*	*14.34 ± 0.74*	*29.06 ± 0.65*
	*Oxygenated sesquiterpenes*		*0.83 ± 0.07*	*5.13 ± 0.17*	*3.30 ± 0.30*	*7.57 ± 0.18*
	***Total identified***		***99.89 ± 0.05***	***41.71 ± 2.18***	***95.56 ± 0.41***	***98.57 ± 0.28***

^a^ Linear retention index on ZB-5MS column; ^b^ Expressed as the mean percentage area extracted from the GC-MS chromatograms of three repeated analyses; * standard injection: the major volatile compounds are in bold; sample codes as in Table 1.

**Table 3:** LC-HRMS/MS profile (tentative annotation) of extracts obtained from rosemary (raw materials, post-distillation materials, or post-supercritical CO_2_ materials).

No	Compound	Class	T_R_ (min)	[M–H]^–^ (*m*/*z*)	MF	HRMS/MS (*m*/*z*)	Sample	Ref.
1	Quinic acid *	Organic acid	1.9	191.0599	C_7_H_12_O_6_	173.0492, 127.0427, 111.0470	RTE, RWE, RSE, RSC1, RSC2, RSC3	[27]
2	Danshensu	Phenolic acid	5.4	197.0451	C_9_H_10_O_5_	179.0355, 151.0408, 135.0455, 123.0452	RTE, RWE, RSE, RSC3	[2]
3	Hydroxybenzoic acid *	Phenolic acid	9.6	137.0243	C_7_H_6_O_3_	108.0218	RWE, RSE	[25]
4	Caffeic acid *	Phenolic acid	13.9	179.0359	C_9_H_8_O_4_	135.0450, 107.0503	RTE, RWE, RSE, RSC1, RSC2, RSC3	[28]
5	Gallocatechin *	Flavonoid	24.3	305.0773	C_15_H_14_O_7_	225.118	RTE, RWE, RSE, RSC1, RSC2, RSC3	[27]
6	Rosmarinic acid *	Phenolic acid	26.7	359.0855	C_18_H_16_O_8_	197.0491, 179.0380, 161.0272, 135.0475	RTE, RWE, RSE, RSC1, RSC2, RSC3	[28]
7	Luteolin-*O*-glucuronide	Flavonoid	28.3	461.0730	C_21_H_18_O_12_	285.0471, 151.0064, 133.0320	RTE, RWE, RSE, RSC1, RSC2, RSC3	[27]
8	Salvianolic acid A	Phenolic acid	30.1	493.1190	C_26_H_22_O_10_	313.0757, 295.0646, 197.0471, 185.0264	RTE, RWE, RSE, RSC1, RSC2, RSC3	[26]
9	Luteolin-*O*-acetylglucuronide I	Flavonoid	31.2	503.0837	C_27_H_20_O_10_	285.0486, 133.0326	RTE, RWE, RSE, RSC1, RSC2, RSC3	[27]
10	Luteolin-*O*-acetylglucuronide II	Flavonoid	31.9	503.0839	C_23_H_20_O_13_	285.0372, 151.0023, 133.0283	RTE, RWE, RSE, RSC1, RSC2, RSC3	[27]
11	Cirsimaritin	Flavonoid	33.0	313.0710	C_17_H_14_O_6_	161.0241, 151.0388, 133.0288	RSE	[27]
12	Ladanein	Flavonoid	34.0	313.0707	C_17_H_14_O_6_	161.0239, 133.0293	RWE, RSE	[25]
13	Rosmanol	Diterpene	36.1	345.1694	C_20_H_26_O_5_	301.1818, 283.1711	RTE, RWE, RSE, RSC1, RSC2, RSC3	[27]
14	Epirosmanol	Diterpene	37.4	345.1706	C_20_H_26_O_5_	301.1802, 283.1706, 268.1467, 227.1078	RTE, RWE, RSE, RSC1, RSC2, RSC3	[27]
15	Isorosmanol	Diterpene	38.6	345.1707	C_20_H_26_O_5_	301.1828, 283.1725, 268.1478, 227.1087	RTE, RWE, RSE, RSC1, RSC2, RSC3	[27]
16	Genkwanin	Flavonoid	40.5	283.0616	C_16_H_12_O_5_	268.0367, 240.0416, 151.0030	RTE, RWE, RSE, RSC1, RSC2, RSC3	[27]
17	Epiisorosmanol	Diterpene	42.1	345.1730	C_20_H_26_O_5_	301.1806, 285.1507	RTE, RSE, RSC1, RSC2, RSC3	[27]
18	Epirosmanol methyl ether	Diterpene	44.0	359.1865	C_21_H_28_O_5_	315.1961, 300.1733, 283.1707	RTE, RSE, RSC1, RSC2, RSC3	[28]
19	Methoxyrosmanol	Diterpene	45.3	359.1883	C_21_H_28_O_5_	315.1982, 300.1743, 283.1718	RTE, RSE, RSC1, RSC2, RSC3	[28]
20	Carnosol	Diterpene	46.0	329.1768	C_22_H_26_O_4_	314.1506, 299.1286, 271.0977	RTE, RWE, RSE, RSC1, RSC2, RSC3	[27]
21	Rosmadial I	Diterpene	47.7	343.1549	C_20_H_24_O_5_	315.1621, 299.1679, 287.1673	RTE, RSE, RSC1, RSC2, RSC3	[28]
22	Rosmadial II	Diterpene	48.5	343.1558	C_20_H_24_O_5_	299.1665, 271.1716	RTE, RSE, RSC1, RSC2, RSC3	[28]
23	Carnosic acid I	Diterpene	50.1	331.1908	C_22_H_28_O_4_	287.2095, 244.1529	RTE, RWE, RSE, RSC1, RSC2, RSC3	[27]
24	Carnosic acid methyl ester	Diterpene	51.5	345.2064	C_21_H_30_O_4_	301.2225, 286.2012, 271.1777	RTE, RWE, RSE, RSC1, RSC2, RSC3	[27]
25	Carnosic acid II	Diterpene	52.1	331.1906	C_22_H_28_O_4_	287.2033, 244.1523	RTE, RSE, RSC1, RSC2, RSC3	[27]

MF, molecular formula; * Confirmed by standard; sample codes as in Table 1.

**Table 4:** Total phenolic and flavonoid content of rosemary extracts obtained from raw post-distillation, or post-SC-CO_2_ materials.

Extracts	TPC (mg GAE/g)	TFC (mg RE/g)
**RTE**	99.36 ± 1.17 ^c^	27.46 ± 0.19 ^c^
**RSE**	57.68 ± 0.54 ^d^	19.86 ± 0.13 ^e^
**RWE**	108.10 ± 0.26 ^a^	30.41 ± 0.17 ^b^
**RSC1**	97.68 ± 5.95 ^b^	27.99 ± 0.70 ^c^
**RSC2**	98.02 ± 1.46 ^b^	32.58 ± 0.11 ^a^
**RSC3**	66.65 ± 4.11 ^c^	21.63 ± 0.15 ^d^

Values are reported as mean ± SD of three parallel measurements: TPC: Total phenolic content; TFC: Total flavonoid content; GAE: Gallic acid equivalent; RE: Rutin equivalent. Different letters indicate significant differences among the extracts from each species (*p* < 0.05); sample codes as in Table 1.

**Table 5:** Antimicrobial activity of rosemary extracts obtained from raw, post-distillation or post-SC-CO_2_.

Microorganism	REO	RC1	RC2	RC3	RTE	RSE	RWE	RSC1	RSC2	RSC3	Control
	MIC [mg/L]										
**Gram-positive bacteria**											**Vancomycin**
*Staphylococcus aureus* ATCC 25923	>1000	31.3	250	1000	31.3	15.6	>1000	15.6	31.3	31.3	0.98
*Staphylococcus aureus* ATCC BAA-1707 *	>1000	62.5	1000	>1000	125	62.5	>1000	125	125	125	0.98
*Staphylococcus epidermidis* ATCC 12228	>1000	31.3	250	1000	62.5	62.5	>1000	62.5	125	125	0.98
*Micrococcus luteus* ATCC 10240	>1000	15.6	250	250	62.5	31.3	250	31.3	62.5	62.5	0.12
*Enterococcus faecalis* ATCC 29212	>1000	125	>1000	1000	125	31.3	>1000	125	62.5	62.5	1.95
*Bacillus cereus* ATCC 10876	>1000	62.5	250	1000	62.5	62.5	>1000	125	125	125	0.98
**Gram-negative bacteria**											**Ciprofloxacin**
*Salmonella* Typhimurium ATCC 14028	>1000	>1000	>1000	>1000	1000	1000	>1000	1000	>1000	>1000	0.061
*Escherichia coli* ATCC 25922	>1000	>1000	>1000	>1000	1000	1000	>1000	1000	>1000	>1000	0.015
*Proteus mirabilis* ATCC 12453	>1000	>1000	>1000	>1000	250	250	1000	250	250	500	0.030
*Klebsiella pneumoniae* ATCC 13883	>1000	>1000	>1000	>1000	>1000	>1000	>1000	>1000	>1000	>1000	0.122
*Pseudomonas aeruginosa* ATCC 9027	>1000	>1000	>1000	>1000	1000	1000	>1000	1000	1000	1000	0.488
**Yeasts**											**Nystatin**
*Candida glabrata* ATCC 2091	1000	2000	2000	1000	2000	1000	>2000	1000	2000	2000	0.48
*Candida albicans* ATCC 102231	2000	>2000	1000	1000	1000	1000	>2000	1000	1000	1000	0.24
*Candida parapsilosis* ATCC 22019	1000	2000	500	500	500	250	1000	250	250	125	0.24

* Methicillin-resistant *Staphylococcus aureus* (MRSA) strain; MIC, minimum inhibitory concentration; sample codes as in Table 1.

**Table 6:** Antioxidant properties of rosemary extracts obtained from raw, post-distillation, or post-SC-CO_2_ materials.

Extracts	DPPH (mg TE/g)	ABTS (mg TE/g)	CUPRAC (mg TE/g)	FRAP (mg TE/g)	MCA (mg EDTAE/g)	PBD (mg TE/g)
**REO**	3.70 ± 0.43 ^h^	32.05 ± 0.12 ^f^	33.49 ± 0.61 ^g^	26.34 ± 1.00 ^g^	na	18.08 ± 0.11 ^a^
**RC1**	38.89 ± 1.66 ^f^	55.61 ± 0.69 ^e^	68.15 ± 3.73 ^f^	47.04 ± 1.49 ^f^	na	4.04 ± 0.16 ^b^
**RC2**	10.63 ± 0.34 ^g^	20.83 ± 0.17 ^f^	33.07 ± 1.07 ^g^	21.03 ± 0.41 ^gh^	na	3.65 ± 0.32 ^bc^
**RC3**	2.53 ± 0.61 ^h^	6.30 ± 0.29 ^g^	17.76 ± 0.39 ^h^	11.24 ± 0.12 ^h^	na	3.34 ± 0.35 ^cd^
**RTE**	144.17 ± 1.93 ^c^	155.03 ± 7.44 ^c^	312.61 ± 6.76 ^b^	197.87 ± 10.97 ^ab^	1.60 ± 0.28 ^d^	2.10 ± 0.02 ^g^
**RSE**	48.45 ± 0.11 ^e^	69.19 ± 0.02 ^e^	166.92 ± 2.57 ^e^	80.96 ± 1.92 ^e^	na	1.41 ± 0.01 ^h^
**RWE**	164.08 ± 4.50 ^b^	179.76 ± 5.43 ^b^	396.28 ± 7.48 ^a^	205.38 ± 3.48 ^a^	8.63 ± 0.45 ^a^	2.33 ± 0.01 ^fg^
**RSC1**	158.77 ± 4.48 ^b^	157.31 ± 4.54 ^c^	269.84 ± 6.85 ^c^	173.25 ± 3.50 ^c^	5.34 ± 0.70 ^b^	2.91 ± 0.08 ^de^
**RSC2**	173.49 ± 1.20 ^a^	202.19 ± 10.73 ^a^	317.00 ± 4.96 ^b^	192.31 ± 5.22 ^b^	3.13 ± 0.18 ^c^	2.72 ± 0.19 ^ef^
**RSC3**	94.77 ± 0.92 ^d^	105.27 ± 0.69 ^d^	182.52 ± 6.74 ^d^	104.11 ± 4.03 ^d^	1.56 ± 0.23 ^d^	2.12 ± 0.14 ^g^

Values are reported as mean ± SD of three parallel measurements. TE: Trolox equivalent. EDTAE: EDTA equivalent; na: not active; Different letters indicate significant differences among the extracts/essential oils from each species (*p* < 0.05); sample codes as in Table 1.

**Table 7:** Enzyme-inhibitory properties of rosemary extracts obtained from raw, post-distillation, or post-SC-CO_2_ materials.

Extracts	AChE (mg GALAE/g)	BChE (mg GALAE/g)	Tyrosinase (mg KAE/g)	Amylase (mmol ACAE/g)	Glucosidase (mmol ACAE/g)
REO	3.05 ± 0.24 ^b^	1.65 ± 0.19 ^b^	59.23 ± 2.80 ^a^	0.39 ± 0.03 ^a^	na
RC1	na	1.76 ± 0.08 ^b^	44.59 ± 0.60 ^b^	0.33 ± 0.02 ^b^	1.20 ± 0.03 ^a^
RC2	3.53 ± 0.18 ^a^	2.88 ± 0.35 ^a^	42.23 ± 0.59 ^bc^	0.27 ± 0.01 ^cd^	1.17 ± 0.02 ^a^
RC3	3.65 ± 0.14 ^a^	3.01 ± 0.13 ^a^	38.13 ± 0.53 ^d^	0.24 ± 0.01 ^d^	0.94 ± 0.01 ^d^
RTE	1.36 ± 0.11 ^c^	na	23.81 ± 0.47 ^e^	0.08 ± 0.01 ^e^	1.04 ±0.01 ^bc^
RSE	1.12 ± 0.06 ^c^	na	20.81 ± 0.07 ^e^	0.07 ± 0.01 ^e^	1.24± 0.02 ^a^
RWE	1.12 ± 0.03 ^c^	na	22.71 ± 0.31 ^e^	0.06 ± 0.01 ^e^	0.96 ± 0.01 ^cd^
RSC1	3.79 ± 0.06 ^a^	na	39.38 ± 2.06 ^cd^	0.32 ± 0.01 ^b^	1.08 ± 0.06 ^b^
RSC2	3.09 ± 0.07 ^b^	na	37.84 ± 0.46 ^d^	0.29 ± 0.01 ^bc^	0.78 ± 0.05 ^e^
RSC3	3.80 ± 0.18 ^a^	0.54 ± 0.04 ^c^	37.10 ± 0.52 ^d^	0.27 ± 0.01 ^cd^	0.88 ± 0.03 ^d^

Values are reported as mean ± SD of three parallel measurements. GALAE: Galanthamine equivalent; KAE: Kojic acid equivalent; na: not active; Different letters indicate significant differences among the extracts/essential oils from each species (*p* < 0.05); sample codes as in Table 1.

## Data Availability

Data is contained within the article.
